# Synthesis of camphor thiazole derivates from *Dryobalanops aromatica* and its bioactivity as antioxidants and antidiabetes against alpha glucosidase enzymes

**DOI:** 10.1016/j.mex.2023.102429

**Published:** 2023-10-10

**Authors:** Antonius Herry Cahyana, Gusman Santika, Kandarpa Phukan

**Affiliations:** a Department of Chemistry, Faculty of Mathematics and Natural Sciences, Universitas Indonesia, Depok, West Java 16424, Indonesia; b P.G. Department of Chemistry, Handique Girls’ College, Guwahati, Assam 781001, India

**Keywords:** Camphor thiazole derivates synthesize, *Dryobalanops aromatica*, Camphor, Thiazole, Antioxidant, Antidiabetic

## Abstract

Camphor is synthesized from the Sumatran camphor plant (*Dryobalanops aromatica*) in previous experiments. It can be synthesized with thiosemicarbazide, ethy-2‑chloro acetoacetate, and sodium acetate (catalyze) to form camphor derivate with thiazole ring structure. Hydrazine and phenylhydrazine were both used to make the thiazole ring variations. All the compounds were purified by recrystallization method and characterized by TLC, FTIR, UV–vis, and LC-MS*.* Camphor thiazole (Product 1), camphor thiazole hydrazine (Product 2), and camphor thiazole phenylhydrazine (Product 3) were successfully synthesized with%yields of 73.24 %; 77.36 %; and 72.91 % respectively. Furthermore, their antioxidant activity was measured using the DPPH free radical method. Product 2 had the strongest antioxidant activity with IC_50_ value of 6.93 ppm. The antidiabetic activity was measured using the α-glucosidase enzyme. This indicated that product 1 had the best inhibitory activity against the α-glucosidase enzyme with IC_50_ values of 869.06 ppm.

• We developed an alternative method to utilize camphor extracted from the *D. aromatica* plant to be used as an alternative medicinal ingredient related to antioxidants and antidiabetes.
• All products were successfully synthesized and have the potential to be used as antioxidants with an IC_50_ value of 6.93 ppm for Product 2 and as antidiabetics by means of an α-glucosidase inhibitor with an IC_50_ value of 869.06 ppm for Product 1.

## Graphical abstract

**Figure:**
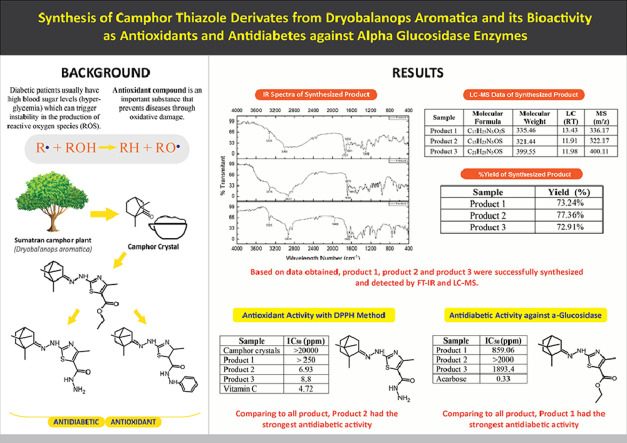

Specifications Table

**Table:** 

Subject area:	Chemistry
More specific subject area:	Organic synthesize
Name of your method:	Camphor thiazole derivates synthesize
Name and reference of original method:	Not available
Resource availability:	Not available

## Method details

### Background

The structure of natural product compounds is commonly used as a precursor to the development of potential new drugs by modifying their structure. For example, as an antioxidant compound which is an important substance for the body to prevent disease due to oxidative damage in the body that can cause chronic disease. In addition, diabetes mellitus is also a chronic, life-threatening disorder. This disease causes carbohydrate and lipid metabolism not to be regulated properly by the hormone insulin, increasing blood glucose levels [1]. Heterocyclic compounds are reported to have good antioxidant activity, antibacterial activity, and anticancer activity [2]. According to a study conducted by Singh, compounds with heterocyclic rings also have potential as anti-diabetic substances [3].

In general, *Dryobalanops aromatica* oil extracts contain borneol content up to 26.02 %, in the form of oil and 92.70 % in the form of crystals [4]. So this plant is suitable for producing borneol. Borneol can be oxidized to camphor so that the ketone group in camphor can be used to add a thiazole heterocyclic ring to the structure to increase its bioactivity. Thiazole (C_3_H_7_NS) is a five-ring heterocyclic compound that has an important role in the pharmaceutical field [5]. Thiazole compounds were obtained through the reaction of thiazole Hantzsch formation, namely the reaction between thiourea and alpha halocarbonyl to produce thiazoles with good yields [6]. Thiazoles can be combined with the camphor structure through the formation of an imine group reaction. So based on the explanation that has been described, it is necessary to synthesize camphor thiazole derivatives and through this research, the synthesis of camphor thiazole derivatives from *D. aromatica* can be carried out and used as antioxidants and antidiabetics.

## Experimental

### Equipment and analytical instruments

All glass apparatus is cleaned and oven-dried. Homogeneity of the reaction products was verified by silica gel-coated thin layer chromatography (TLC) plate. Functional groups of product structure were identified by Shimadzu IR Prestige 21 FT-IR Spectrometer in the range 4000–400 cm^−1^. Maximum wavelength (λ) of a compound identified by Shimadzu UV-2450 UV–vis Spectrophotometer in the range of 200–800 nm. Chromatograms and mass spectra were detected using Liquid Chromatography Mass Spectrometry (Waters Xevo G2-S Qtof, ACQUITY UPLC®HSS C18 H-Class System). Stuart™ Analog Melting Point Apparatus was used to identify the melting point of the compound.

### Chemical reagents

Camphor (synthesized from *D. aromatica*), sodium acetate (CH_3_COONa) catalyze, thiosemicarbazide, ethyl-2-chloroacetoacetate, ethanol, chloroform, aquadest, n-hexane, ethyl acetate, 2,2-diphenyl-1-picrylhydrazyl (DPPH), 4-nitrophenyl α-d-glucopyranoside, α-Glucosidase Enzyme (from *Bacillus stearothermophilus*), phosphate buffer, and sodium carbonate (Na_2_CO_3_) solution.

### Camphor thiazole synthesize method

Camphor (1 mmol), ethyl-2‑chloro acetoacetate (1 mmol), and thiosemicarbazide (1 mmol) were dissolved in 10 mL ethanol. A sodium acetate catalyst (0.02 mmol) was added as a catalyze. Reflux for 10 h at 78 °C in constant stirring then cooled at room temperature. Leave it overnight. Pour the mixture into iced aquadest on a beaker glass, and mix it until a precipitate formed then separated through a filtration process. Recrystallize with ethanol to purify the desired product (camphor thiazole, *product 1*). To add variety to the structure, camphor thiazole (1 mmol) and hydrazine (*or* Phenylhydrazine, 1 mmol) were dissolved in 10 mL ethanol. Reflux for 4 h at 70 °C in constant stirring. After the reaction, the mixture was evaporated with dry air flow. Then recrystallize the precipitate with ethanol to purify the desired product (camphor thiazole hydrazine, *or* product 2, and camphor thiazole hydrazine*, or* product 3*)*.

### Antioxidant activity assay

The antioxidant activity was measured using 2,2-diphenyl-1-picrylhydrazil (DPPH) as a free radical. A total sample solution with various concentrations at a volume of 2.0 mL was added with 2 mL of the DPPH solution (4 mg in 100 mL of ethanol). Left it in a dark room for 30 min at room temperature. UV–vis spectrophotometer was used to read the adsorption wavelength at 517 nm.

### Antidiabetic activity assay

The mixture consisted of 50 µL 0.1 M phosphate buffer solution, 25 µL 0.5 mM 4-nitrophenyl α-d-glucopyranoside (dissolved in phosphate buffer solution), 10 µL of test sample solution, and 25 µL of α-glucosidase solution (1 mg/mL stock solution in phosphate buffer). This reaction occurs at pH 7.0. Incubate at 37 °C for 30 min. Then reaction stopped by adding 100 µL of 0.2 M sodium carbonate solution. Enzymatic hydrolysis of the substrate was monitored by the amount of *p*-nitrophenol released in the reaction mixture at 410 nm using a microplate reader.

### Analytical calculation

The percentage of inhibition was measured to know inhibition activity in various concentrations. The percentage of inhibition value for every concentration of the sample in antioxidant and antidiabetic assay were calculated with the following equation:

*%*Inhibition = ((Abs_blanko_ – Abs_sample_)/Abs_blanko_) x 100 %

IC_50_ was calculated to know the concentration that has 50 % inhibition activity.

## Results and discussion

### Synthesize of camphor thiazole

**Fig. 1:**
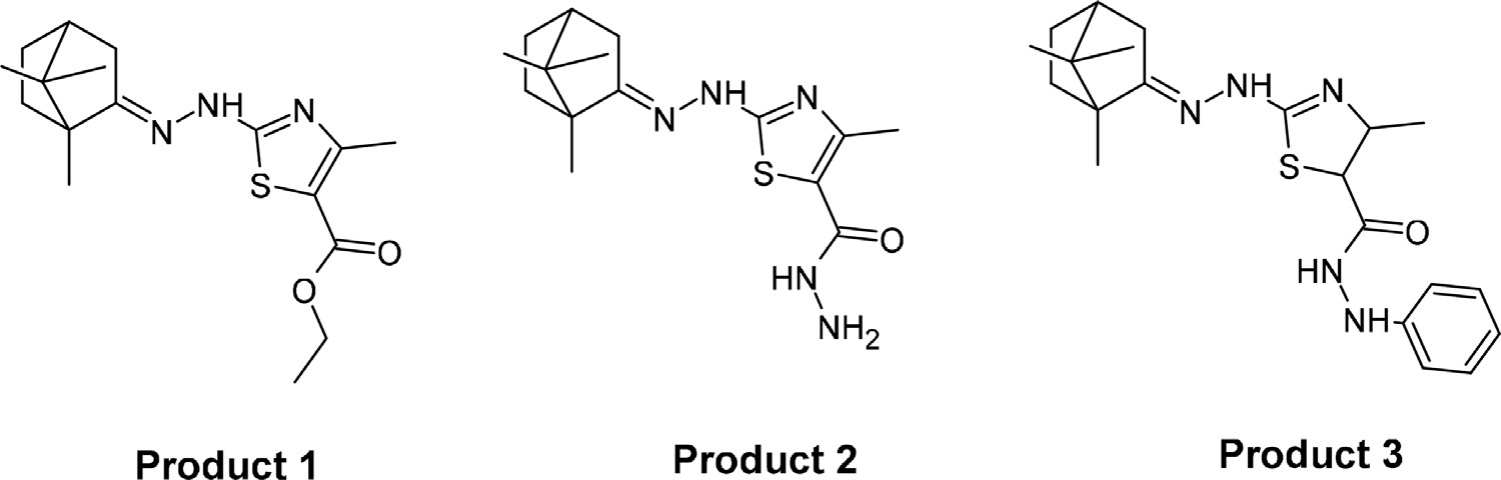
Structure of synthesized product.

In this study, the camphor used as the main precursor in the synthesis map was a camphor compound that was oxidized from lime oil crystals obtained from plant oil *D. aromatica* oil. This is done to utilize the potential of natural materials in Indonesia and increase their usefulness. The structure of the compound that has been successfully synthesized can be seen in Fig. 1.

**Scheme 1:**
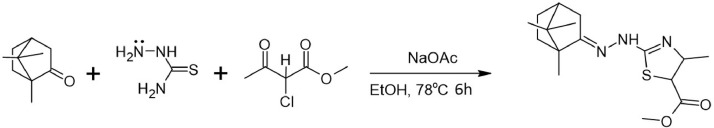
Synthesize reaction of Product 1.

The reaction process for the synthesis of product 1 (Scheme 1) took place with a change in the color of the mixture from clear yellowish to red. Tests using TLC were carried out to ensure that the compound had been formed. The eluent used in the TLC test for this compound was ethyl acetate (EA): n-hexane (Hex) with a ratio of 4:1. The Rf values ​​of product 1 compared with the Rf values of thiosemicarbazide were 0.78 and 0.38, respectively. The difference in Rf between the precursor and the product indicates that the compound product 1 has been formed. In this experiment, the percentage yield obtained was 73.24 % of the expected result. Based on the melting point test, it is known that the compound formed has a melting point at 210–214 °C. Product 1 was detected in chromatogram at retention time of 13.43 min. In the mass spectrum (TOF MS ES+) the detected value of [M + H]^+^ is 336.1751 u. This is in accordance with theoretical calculations, where the theoritical value of [M + H]^+^ of this compound is 322.1745.

**Scheme 2:**
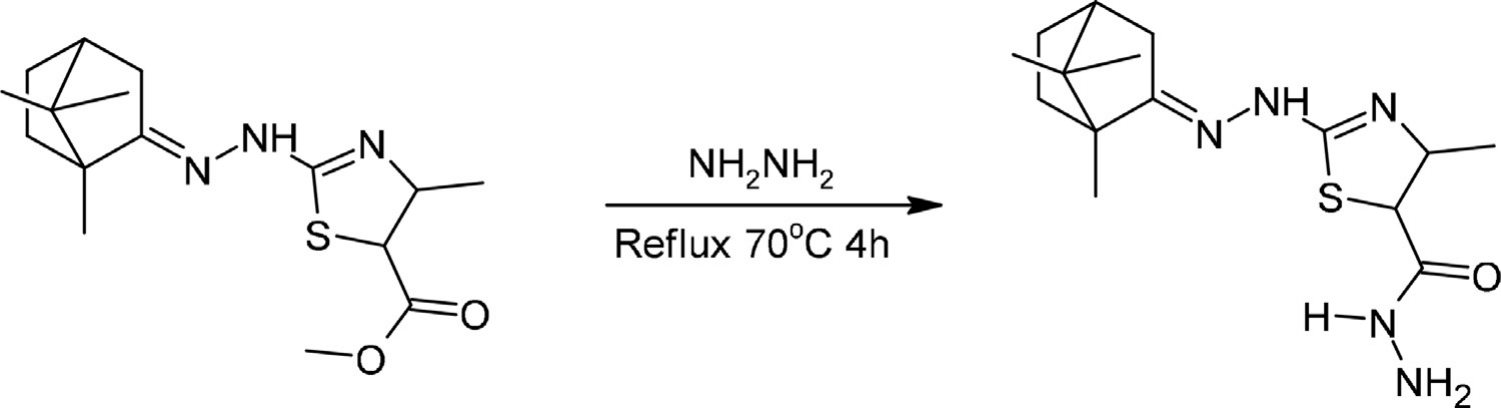
Synthesize reaction of Product 2.

The result of product 2 formation reaction (Scheme 2) can be observed by a change in the color of the mixture to a slightly brownish fading. The reaction results were obtained in the form of a pale yellowish orange solid. The TLC test was carried out using EA: Hex eluent in a ratio of 3:1. The Rf values of product 2 compared to the precursors of product 1 were 0.44 and 0.48, respectively. The difference in Rf between the precursor and the product indicates that product 2 has been formed. Based on this experiment, the percentage yield obtained was 77.36 %. It is known that this compound has a melting point of 203–206 °C. Product 2 was detected in the chromatogram at retention time of 11.91 min. In the mass spectrum (TOF MS ES+) the detected value of [M + H]^+^ is 322.1764 u. This is in accordance with theoretical calculations, where the theoretical value of [M + H]^+^ of this compound is 322.1701.

**Scheme 3:**
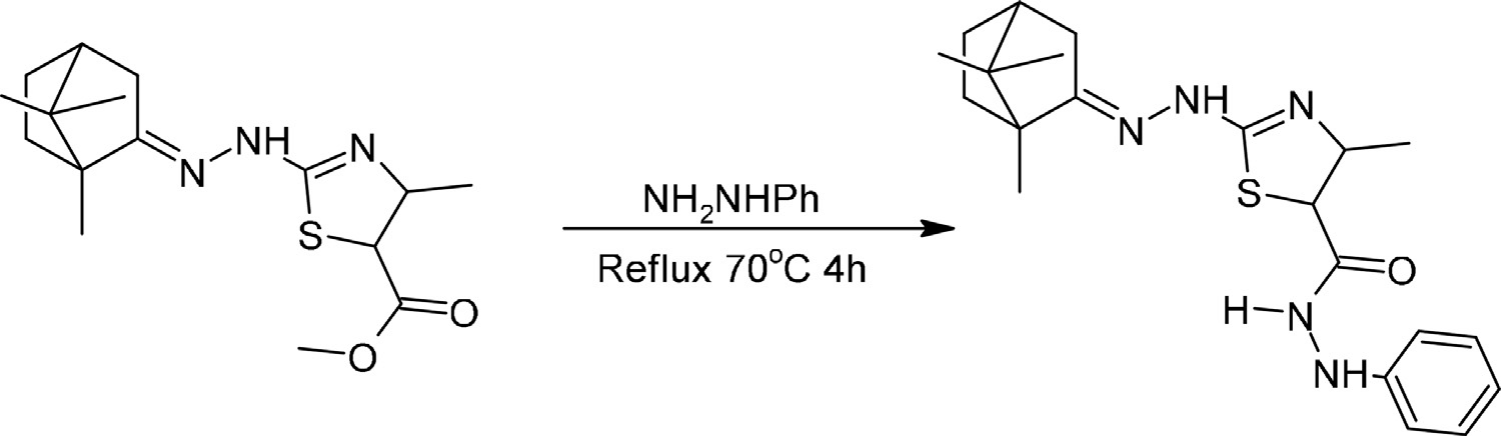
Synthesize reaction of Product 3.

The results of the reaction for the formation of product 3 (Scheme 3) can be observed when the color of the mixture becomes dark red. The TLC test was carried out using EA: Hex eluent with a ratio of 1:1. The Rf values of product 3 compared to the precursors of product 1 were 0.88 and 0.80, respectively. The difference in Rf between the precursor and the product indicates that the compound product 3 has been formed. Based on this experiment, the reaction product was obtained in the form of a solid with a 72.91 % yield. This compound has a melting point of 236–240 °C. Product 3 was detected by the chromatogram at retention time of 11.98 min. In the mass spectrum (TOF MS ES+) it is known that the detected value of [M + H]^+^ is 400.2115 u. This is in accordance with theoretical calculations, where the theoritical value of [M + H]^+^ of this compound is 400.2171.

Complete data of the physiochemistry of the compounds that were successfully synthesized shown in Table 1.

**Table 1:** Physicochemical data of synthesized product.

Product	Molecular formula	Molecular weight	Melting point	TLC (Rf) EA: Hex	LC (RT)	MS (*m/z*)	% Yield
1	C_17_H_25_N_3_O_2_S	335.46	210–214 °C	0.78 (4:1)	13.43	336.17	73.24 %
2	C_15_H_23_N_5_OS	321.44	203–206 °C	0.44 (3:1)	11.91	322.17	77.36 %
3	C_21_H_29_N_5_OS	399.55	236–240 °C	0.88 (1:1)	11.98	400.11	72.91 %

**Fig. 2:**
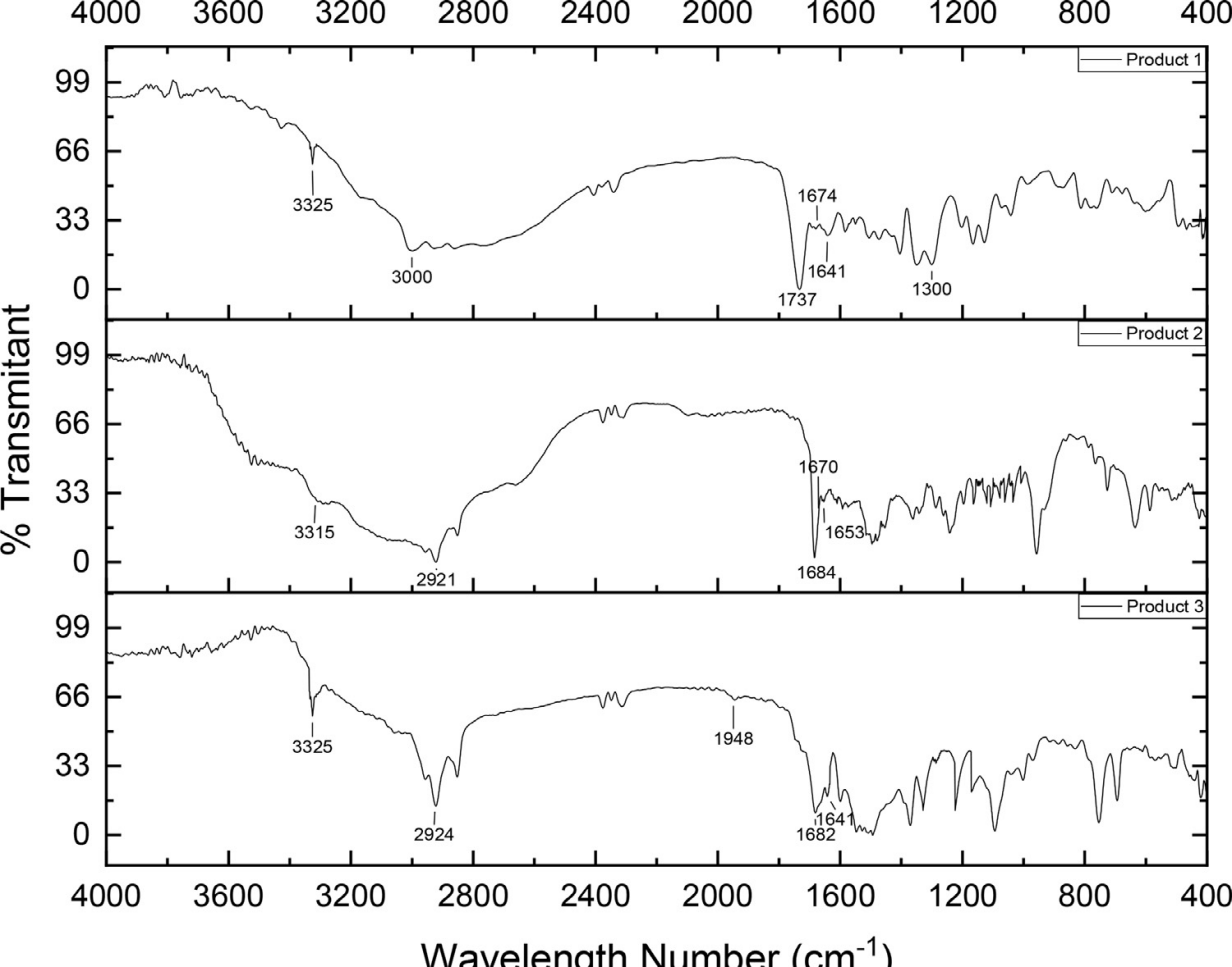
IR Spectra of synthesized product.

The product that has been successfully formed was characterized using the Fourier Transform InfraRed (FTIR) Spectrometer instrument to identify the functional groups contained in the structure of the compound. The IR spectrum can be seen in Fig. 2 and the detected wave numbers are summarized in Table 2.

**Table 2:** Detected wavelength number in IR spectra of synthesized product.

Functional group	Wavelength number (cm^−1^)		
	Product 1	Product 2	Product 3
N—H *sec a*mine	3325	3315	3325
C—H alkane	3000	2921	2924
C=O ester	1737	–	–
C=C alkene	1674	1670	–
C=N	1641	1653	1641
C—O ester	1300	–	–
C=O *sec* amide	–	1684	1682
Aromatic benzene	–	–	1948

### Anti-oxidant activity

In this study, antioxidant activity was tested to observe the effect of the tested samples on the free radical compound DPPH (1,1-diphenyl-2-picrylhydrazyl). The results of the antioxidant activity test of the tested samples can be seen in Table 3.

**Table 3:** Antioxidant activity of synthesized product with DPPH method.

Sample	IC_50_
*D. aromatica* oil	4293 ppm
*D. aromatica* crystals	16,819 ppm
Camphor crystals	>20,000 ppm
Product 1	>250 ppm
Product 2	6.93 ppm
Product 3	8.8 ppm
Vitamin C	4.72 ppm

Based on the antioxidant activity testing that has been carried out on each sample, it can be seen that the modification of camphor with thiazole hydrazine/phenylhydrazine has good antioxidant activity against DPPH free radicals and has the potential to be used as an antioxidant compound, although the IC_50_ value of each of these compounds has not been able to compete with the IC_50_ value of Vitamin C compound which has an IC_50_ value of 4.72 ppm.

### Anti-diabetic activity

Antidiabetic activity of the compounds that have been successfully synthesized was carried out *in vitro* against the α-glucosidase enzyme. The results of the measurement of the inhibitory activity of the sample substance against the α-glucosidase enzyme can be seen in Table 4.

**Table 4:** Antidiabetic activity of synthesized product agains alpha glucosidase enzyme.

Sample	IC_50_
Camphor thiazole	859.06
Camphor thiazole hydrazine	>2000
Camphor thiazole phenylhydrazine	1893.4
Acarbose	0.33

Based on the experiments that have been carried out, it can be seen that the higher the concentration value of the added sample will increase the inhibitory activity. Based on calculations, the IC_50_ value of camphor thiazole is 859.06 ppm which is higher than camphor thiazole hydrazine and camphor thiazole phenylhydrazine which have IC_50_ values of 1893.40 ppm and >2000 ppm, respectively. This shows that the addition of a hydrazine/phenylhydrazine group that replaces the ester group on the camphor thiazole compound does not increase its activity ability to inhibit the αglucosidase enzyme. Camphor with a thiazole ring has the potential to be used as an antidiabetic compound in inhibiting the activity of the α-glucosidase enzyme, although the IC_50_ value of each compound can still be said to be weaker than the IC_50_ value of the Acarbose compound which has an IC_50_ value of 0.33 ppm.

## Conclusion

The synthesized camphor from *D. aromatica* oil can be used to synthesize camphor thiazole compounds, camphor thiazole hydrazine, and camphor thiazole phenylhydrazine with a yield of 73.24 %; 77.36 %; and 72.91 % respectively. In addition, the synthesized compounds showed antioxidant and antidiabetic activity. Camphor thiazole hydrazine is known to have the strongest antioxidant activity compared to other products with an IC_50_ value of 6.93 ppm. Meanwhile, camphor thiazole compound is known to have the best antidiabetic activity against α-glucosidase enzyme compared to other products with an IC_50_ value of 869.06 ppm.

## Ethics statements

Not applicable.

## CRediT authorship contribution statement

**Antonius Herry Cahyana:** Conceptualization, Methodology, Supervision, Writing – review & editing. **Gusman Santika:** Investigation, Validation, Data curation, Software, Visualization, Writing – original draft. **Kandarpa Phukan:** Supervision.

## Declaration of Competing Interest

The authors declare that they have no known competing financial interests or personal relationships that could have appeared to influence the work reported in this paper

## Data Availability

The authors do not have permission to share data.
